# Low SAR ^31^P (multi‐echo) spectroscopic imaging using an integrated whole‐body transmit coil at 7T

**DOI:** 10.1002/nbm.4178

**Published:** 2019-10-14

**Authors:** Q. van Houtum, D. Welting, W.J.M. Gosselink, D.W.J. Klomp, C.S. Arteaga de Castro, W.J.M. van der Kemp

**Affiliations:** ^1^ University Medical Center Utrecht Utrecht The Netherlands

**Keywords:** fast acquisition, in vivo, MRSI, quantification, RF, SAR, X‐nuclei

## Abstract

Phosphorus (^31^P) MRSI provides opportunities to monitor potential biomarkers. However, current applications of ^31^P MRS are generally restricted to relatively small volumes as small coils are used. Conventional surface coils require high energy adiabatic RF pulses to achieve flip angle homogeneity, leading to high specific absorption rates (SARs), and occupy space within the MRI bore. A birdcage coil behind the bore cover can potentially reduce the SAR constraints massively by use of conventional amplitude modulated pulses without sacrificing patient space. Here, we demonstrate that the integrated ^31^P birdcage coil setup with a high power RF amplifier at 7 T allows for low flip angle excitations with short repetition time (*T*_R_) for fast 3D chemical shift imaging (CSI) and 3D *T*_1_‐weighted CSI as well as high flip angle multi‐refocusing pulses, enabling multi‐echo CSI that can measure metabolite *T*_2_, over a large field of view in the body. *B*_1_^+^ calibration showed a variation of only 30% in maximum *B*_1_ in four volunteers. High signal‐to‐noise ratio (SNR) MRSI was obtained in the gluteal muscle using two fast in vivo 3D spectroscopic imaging protocols, with low and high flip angles, and with multi‐echo MRSI without exceeding SAR levels. In addition, full liver MRSI was achieved within SAR constraints. The integrated ^31^P body coil allowed for fast spectroscopic imaging and successful implementation of the multi‐echo method in the body at 7 T. Moreover, no additional enclosing hardware was needed for ^31^P excitation, paving the way to include larger subjects and more space for receiver arrays. The increase in possible number of RF excitations per scan time, due to the improved *B*_1_^+^ homogeneity and low SAR, allows SNR to be exchanged for spatial resolution in CSI and/or *T*_1_ weighting by simply manipulating *T*_R_ and/or flip angle to detect and quantify ratios from different molecular species.

## Graphical abstract

An integrated custom‐built whole‐body birdcage coil enabled ^31^P MRSI in large volumes without sacrificing space. The *B_1_^+^*‐field uniformity, comparable to 3 T proton MR birdcage coils, allowed using conventional amplitude modulated pulses. This massively reduced SAR constrains compared to adiabatic pulses. The increase in RF pulses per unit of time enabled fast and multi‐echo MRSI in the body at 7 T. Local liver spectra were acquired. Presented setup creates opportunities for new body‐focused ^31^P/X‐nuclei MRS methodologies.

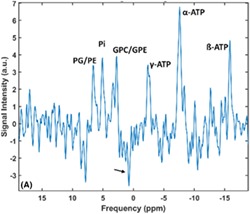

**Abbreviations**
^31^P: phosphorus
AMESING: adiabatic multi‐echo spectroscopic imaging
ATP: adenosine triphosphate
BMI: body mass index
CSI: chemical shift imaging
FID: free induction decay
GPC: glycerophosphocholine
GPE: glycerophosphoethanolamine
MESING: multi‐echo spectroscopic imaging
PC: phosphocholine
PCr: phosphocreatine
PDE: phosphodiester
PE: phosphoethanolamine
P_i_: inorganic phosphate
PME: phosphomonoester
SAR: specific absorption rate
SNR: signal‐to‐noise ratio
*T*_R_: repetition time.

## 1 INTRODUCTION

Phosphorus (^31^P) MRSI provides opportunities to monitor tissue metabolism by measuring specific energy metabolites and phospholipid metabolites. Phosphocreatine (PCr), adenosine triphosphate (ATP) (with α‐, β‐ and γ*‐*resonances) and inorganic phosphate (P_i_) give insight into cell energy metabolism. Decreased PCr/ATP ratios in the heart can be used as diagnostic indicators in systemic diseases such as Type 2 diabetes and obesity.^1^, ^2^, ^3^ P_i_ can be used to calculate tissue pH, as its resonance frequency changes with the acidity of the environment. Phosphomonoesters (PMEs) and phosphodiesters (PDEs) allow assessment of phospholipid metabolism.^4^, ^5^, ^6^ At ultra‐high field (>7 T), the increased signal‐to‐noise ratio (SNR) and increased spectral resolution facilitate the individual detection of PMEs (phosphocholine (PC), phosphoethanolamine (PE)) and PDEs (glycerophosphocholine (GPC), glycerophosphoethanolamine (GPE)).^7^ Enhanced PME to PDE ratios (PC to GPC, PE to GPE) are indicative of proliferation and are often seen in tumor tissue^5^, ^6^, ^8^, ^9^, ^10^, ^11^, ^12^, ^13^ Changes in these ratios during (chemo)therapy are markers of therapy response and take place well before morphological changes can be observed.^14^, ^15^, ^16^

Still, these potential biomarkers are generally monitored to quantify metabolite concentrations or to investigate ratios between different molecular species, thus requiring solely a metabolite density‐weighted signal. From proton MRI it is known that most clinically relevant contrast, when compared with proton density‐weighted MRI, is obtained from *T*_1_ and *T*_2_ weighting. In fact, one study showed that in ^31^P spectra the *T*_2_ relaxation itself may be used as a marker in breast cancer, and another study reported that intra‐mitochondrial and cytosolic P_i_ can be discriminated based on *T*_1_ differences.^17^, ^18^

However, current applications of ^31^P MRS are generally restricted to relatively small volumes such as the brain, breast and calf muscle, as small birdcage or conventional surface coils are used.^4^, ^19^ Conventional surface coils require high energy adiabatic RF pulses to achieve flip angle homogeneity, as inhomogeneous excitation leads to discrepancies in spectra over larger fields of view. Consequently, this can lead to high specific absorption rates (SARs), thus limiting the number of consecutive scans, particularly when considering metabolite relaxation parameter quantifications, fast spectroscopy methods or *T*_1_‐ and *T*_2_‐weighted sequences.

Recent work by van der Kemp et al showed an adiabatic multi‐echo spectroscopic imaging (AMESING) sequence, which included voxel specific *T*_2_ quantification of the different metabolites in the acquired spectrum.^8^, ^18^ This allowed *T*_2_‐weighted SNR enhancement, for an increased metabolite sensitivity, or *T*_2_ information per metabolite. In the AMESING sequence, uniform excitation is achieved using adiabatic half pass RF pulses and homogeneous refocusing with adiabatic BIR‐4 180° pulses, which require high *B*_1_^+^ (~100 μT) and relatively long pulse duration (8 ms). These pulses are therefore SAR demanding and consequently restricted to body surface applications.

Moving to larger fields of view in the body is therefore challenging, as greater *B*_1_ field discrepancies are present with inhomogeneous excitation. Application of larger surface coils and adiabatic pulses would require even more power, which would limit the acquisition even more. In addition, the long *T*_R_ times necessary for sufficient spin relaxation between pulses and for minimization of average SAR result in clinically impractical scan times for a single protocol.

To achieve uniform *B*_1_^+/−^ fields as with conventional ^1^H MRI, small X‐nuclei RF‐birdcage coils for head and extremities allow for diverse pulse sequences and enable numerous contrasts. Indeed even multi‐echo acquisitions in the brain at 7 T are possible within SAR guidelines using these plug‐and‐play devices.^19^

In another recent publication, Löring et al showed an insertable ^31^P birdcage body coil that can produce uniform *B*_1_ fields, thus allowing the use of rectangular RF pulse excitations.^20^ This birdcage coil is wide enough to accommodate the human torso, allowing ^31^P MRSI of the human body, yet occupies space from the bore limiting inclusion of heavy patients and reduces space for receiver coils. Löring et al did show the application of low flip Ernst angle excitations, with accompanying short *T*_R_, which can result in fairly homogeneous excitation fields over the entire spectral bandwidth for in vivo ^31^P MRS at 7 T and acceptable scan times over a larger field of view.

In this work, we demonstrate that the permanent installation of a ^31^P body coil behind the covers of the patient tube, ie without sacrificing patient space, while interfacing to a high power RF amplifier, increases its usability. The reduction in SAR with this body coil allows the use of rectangular and even multiple rectangular composite pulses. Applications on the large gluteal muscle and liver are shown, including low flip angle excitation with short *T*_R_ for fast 3D CSI and 3D *T*_1_‐weighted CSI, as well as high flip angle multi‐refocusing pulses enabling multi‐echo CSI, over a large field of view.

## 2 METHODS

### 2.1 Coil setup

**Figure 1:**
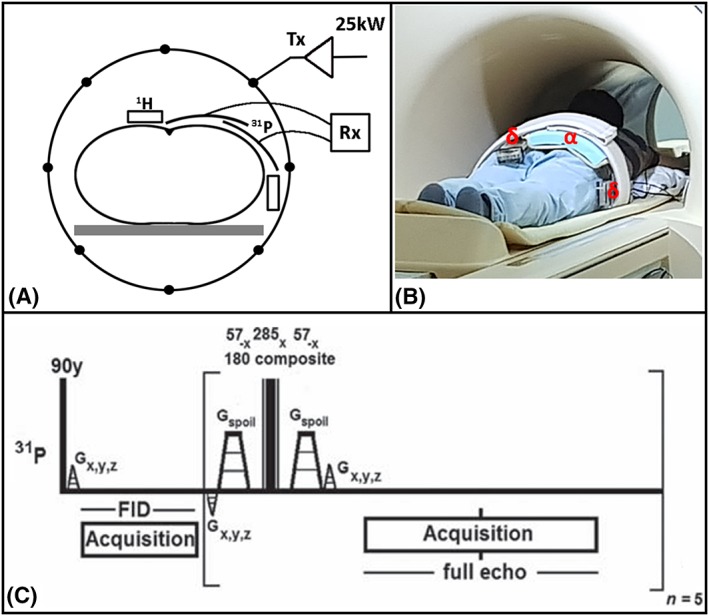
A, schematic diagram of the ^31^P body MRSI setup with the integrated birdcage body coil for transmit represented by the circle including the 25 kW power supply, the ^31^P receiver coils shown by the two arcs, the blocks for the two dipole antennas and the subject centered in the body coil and MR bore. B, photograph of the setup showing a volunteer on the MR bed with the full imaging setup installed including the ^31^P receiver loops (α), the proton dipole antennas (δ) and the noticeable absence (behind patient tube) of the ^31^P body coil, corresponding to the schematic diagram in a. C, the pulse sequence of the MESING protocol with rectangular pulse excitation, refocusing composite block pulses plus the encoding and spoiling gradients

^31^P‐MRSI was performed using an in‐house designed birdcage body coil, permanently integrated into a 7 T MRI system (Philips Healthcare, Best, The Netherlands), with a bore diameter of 60 cm for full body coverage. The coil, tuned at 120 MHz, was interfaced to and driven by a 25 kW RF amplifier for a *B*_1_^+^ field of 15 μT at the center of the bore (Figure 1A).^21^ Two overlapping ^31^P receiver coils (10 × 16 cm^2^) in quadrature mode and two separate fractionated dipole antennas (30 cm) for proton imaging were used in quadrature transceiver mode.^21^ The proton antennas were positioned on the left and right sides of the ^31^P receiver coils, as can be seen in Figure 1A and 1B.

### 2.2 In vitro and in vivo setup

In vitro measurements were made on a body‐sized phantom created from a barrel (diameter 27 cm, height 38 cm) filled with foam, normal saline and a small sphere (diameter 4 cm) filled with a 1 M P_i_ solution. The composition ensured loading matched to a human body for both the ^31^P coils and ^1^H antennas during measurements. For the in vivo measurements four healthy volunteers, three males and one female, with a body mass index (BMI) range of [21.6–26.5], were imaged in prone position with the gluteal muscles at the isocenter of the MR bore. The ^31^P receiver coil and proton imaging setup were placed on the gluteal muscles of the volunteers. One volunteer was imaged in the right decubitus position with the ^31^P receiver coils positioned at liver height, between the bed and the volunteer. The study was approved by the UMC Utrecht Medical Ethical Review Board and all volunteers gave written informed consent.

### 2.3 MR data acquisition

First, a proton image for anatomy localization followed by a *B*_0_ map for image based *B*_0_ shimming were obtained. To make sure that the flip angles were kept similar for all volunteers, a flip angle calibration with the carrier frequency set to PCr (ie set to 0 ppm) was made. The ^31^P *B*_1_^+^ field calibration was done with a non‐localized block pulse sequence with a series of increasing flip angles and a *T*_R_ of 2500 ms, which included gradient spoiling. The zero‐crossing of the signal intensity, marking the actual 180° angle, eg no signal, was used to adjust output power.

Two fast chemical shift protocols using rectangular block pulses with the carrier frequency set to PCr, one with maximized signal intensity for P_i_ using the Ernst angle for P_i_ based on a *T*_1_ of 4300 ms and another at a higher flip angle to increase *T*_1_ weighting, were acquired at an isotropic resolution of 40 mm, matrix size 10 × 6 × 6, 512 acquisition points, bandwidth 8000 Hz, *T*_R_ 150 ms, flip angle, α, 16° and 40°, number of averages 10 and included elliptical *k*‐space sampling resulting in a scan duration of 7 min 3 s.^17^, ^22^

A multi‐echo spectroscopic imaging protocol (MESING), shown in Figure 1C, was used in order to acquire a single free induction decay (FID) by means of a pulse acquire and five full echoes in one *k*‐space step, while *k*‐space data were sampled spherically.^8^, ^19^ The sequence was modified such that the excitation was performed using a rectangular 90° pulse at 15 μT, followed by a composite refocusing made up of three rectangular RF pulses of equal *B*_1_^+^ amplitude and flip angles of 57°_−*x*_, 285°_*x*_ and 57°_−*x*_ for a refocusing bandwidth of 2 ppm. The refocusing part of the sequence is repeated five times to obtain five echoes. The carrier frequency of all pulses was set to P_i_ and PCr for the in vitro and in vivo experiments respectively. The latter was based on the bandwidth of the refocusing pulses and the in vivo ^31^P metabolite with the highest concentration (PCr), which results in increased signal intensity favoring SNR. Both the in vitro and in vivo experiments with MESING were performed with an isotropic resolution of 40 mm with the carrier frequency set to P_i_ and PCr respectively. Other parameters were *T*_R_ 5000 ms, Δ*T*_E_ 45 ms, bandwidth 7800 Hz, matrix 8 × 8 × 6, 256 acquisition points and a scan duration of 21 min 20 s. The in vitro experiment was used to validate the adapted protocol and applicability over a large field of view in vivo.

Liver spectra were acquired using a 3D ^31^P CSI protocol with Hamming‐weighted acquisition at a 15 mm isotropic nominal resolution. The flip angle of 8° and a *T*_R_ of 60 ms were chosen for optimal SNR with the Ernst angle for GPE and GPC assuming a *T*_1_ of around 6000 ms.^23^ The carrier frequency was set to PCr and other CSI parameters were *T*_E_ 0.44 ms, bandwidth 4800 Hz, matrix 12 × 8 × 8, number of sampled averages 80 and 256 acquisition points for a total scan duration of 21 min 48 s.

### 2.4 Data processing

All ^31^P MRSI data were processed in MATLAB 2017 (MathWorks, Natick, MA, USA). Calibrations of the ^31^P *B*_1_^+^ field from all volunteers were summarized by using the peak intensity of PCr of each flip angle dynamic scan obtained for each volunteer after apodization in the time domain with a Gaussian filter of 30 Hz. Data shown were normalized using the maximum signal intensity of each volunteer and the inter‐subject variation was calculated from the variation in the periods of each individual fit per volunteer using

(1) $$\mathrm{SI}=\frac{\sin\alpha \;\left(1-e^{-\frac{T_{R}}{T1}}\right)}{\left(1-e^{-\frac{T_{R}}{T1}}\cos\alpha \right)}.$$

3D multi‐echo spectral data were spatially filtered using a 3D Hamming window, followed by inverse Fourier transformation to the spatial domain. FID and echoes were apodized using a 40 Hz Gaussian filter, and in vivo data were zero filled to double the number of samples thereafter. First order phase correction was applied to the FID by circular shifting the first missing data points resulting from the acquisition delay. *T*_2_ of the metabolites was calculated by fitting a mono‐exponential model using the Levenberg–Marquardt algorithm:

(2) $$\mathrm{SI}=S_{0}\;e^{-\frac{T_{E}}{T_{2}}}.$$

All other 3D CSI data were averaged, spatially filtered using a 3D Hamming window and transformed to the spatial domain by inverse Fourier transformation. The FIDs were apodized in the time domain with a Gaussian filter of 40 Hz and 24 Hz for the gluteal muscle and liver respectively and zero filled to double the number of samples. Phase corrections were applied manually thereafter.

## 3 RESULTS

**Figure 2:**
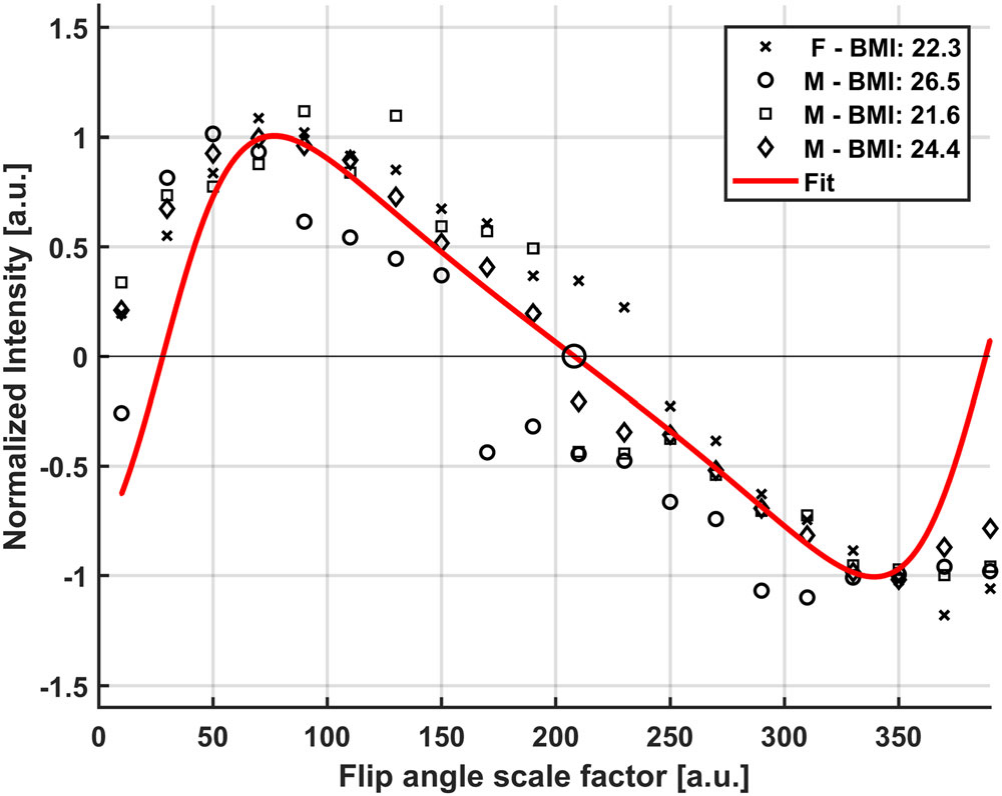
In vivo flip angle series showing the excitation profiles for all four volunteers showing the maximum PCr peak versus each flip angle scale factor. An average inter‐subject variation of 30% was calculated. The BMI and gender of each volunteer is denoted in the legend. The *T*_1_ relaxation effect is apparent from the asymmetry of the 90° versus 180° pulses and mirrored 360° pulse due to not fully relaxed spins. The zero‐crossing of the fit for calculating correct power adjustments is marked by the larger black circle

All subjects fitted well in the ^31^P whole‐body coil setup, as this coil is integrated within the MRI scanner, behind the covers of the bore, providing sufficient space for the dipole transceivers and receive loops as shown in Figure 1A and 1B. The flip angle sweep acquired in four volunteers (different coil load) for *B*_1_^+^ calibration of the ^31^P whole‐body coil is shown in Figure 2; the average variation in maximum *B*_1_ between the volunteers was 30% using 23 kW as peak power. Variation of *B*_1_^+^, as indirectly observed from the flip angle series, remains low despite differences in coil load from the volunteers (BMI range 21–26 kg/m^2^). The power settings were corrected for each volunteer, based on the interpolated zero‐crossing at 180°, which is independent of *T*_1_ relaxation, to achieve the right flip angle on the subsequent acquisitions.

**Figure 3:**
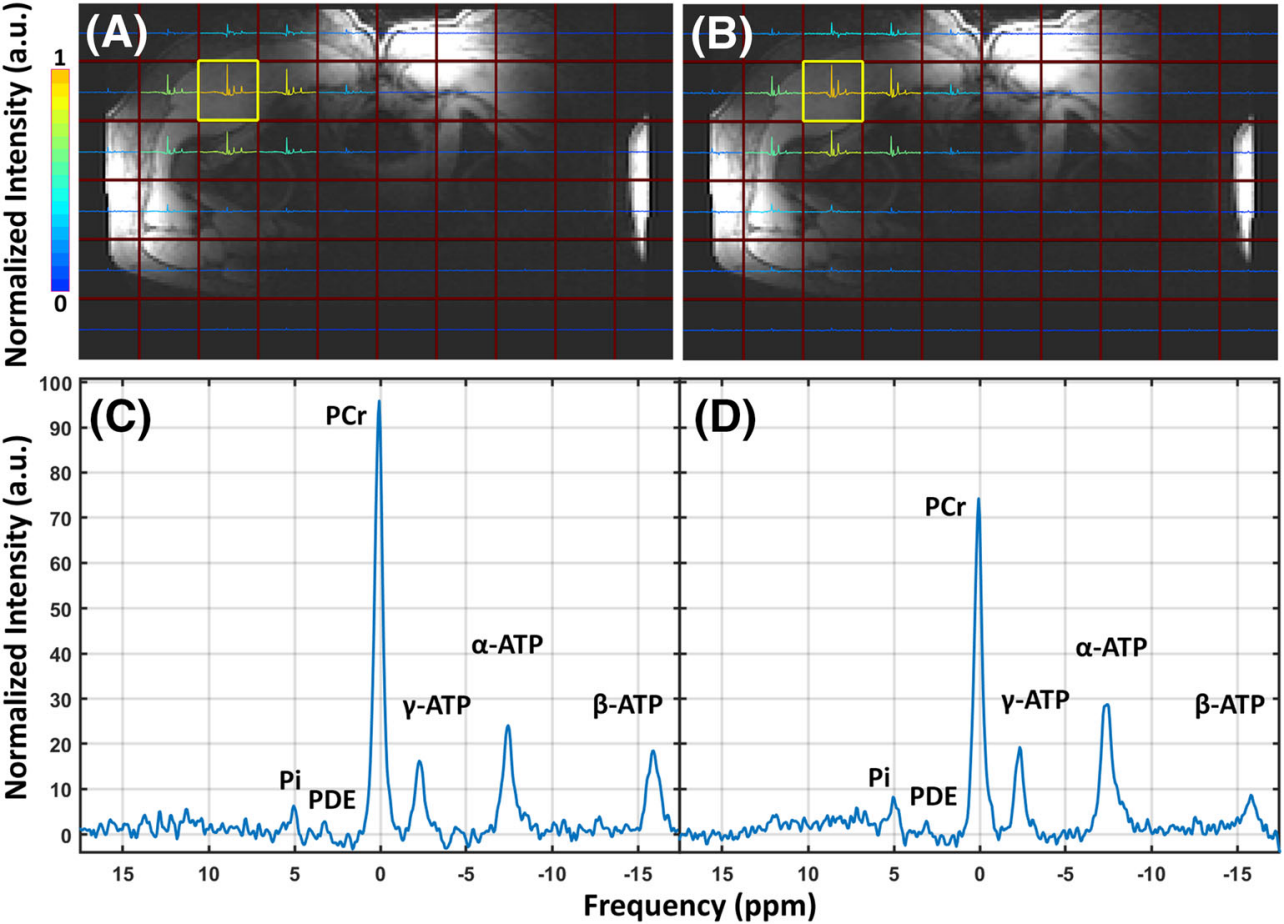
A, B, two spectral images of the in vivo 3D CSI data sets projected on the MR localizer image with a low flip angle (16°) (a) and a high flip angle (40°) (B) with the same *T*_R_ (150 ms). Spectral data are normalized to the maximum signal and the *y*‐axes of all voxels are scaled to the maximum signal in the 2D spectral images. C, D, two in vivo *T*_1_‐weighted spectra of the voxel highlighted by the yellow square in A (C) and the voxel highlighted in B (D), normalized to the noise of each spectrum. Data were acquired using the quadrature mode receiver coil setup in combination with the ^31^P body coil, and *T*_R_ and flip angles were chosen to introduce *T*_1_ weighting. The metabolite peaks of P_i_, the PDEs, PCr and α‐, β‐ and γ‐ATP are labeled in both individual spectra

**Figure 4:**
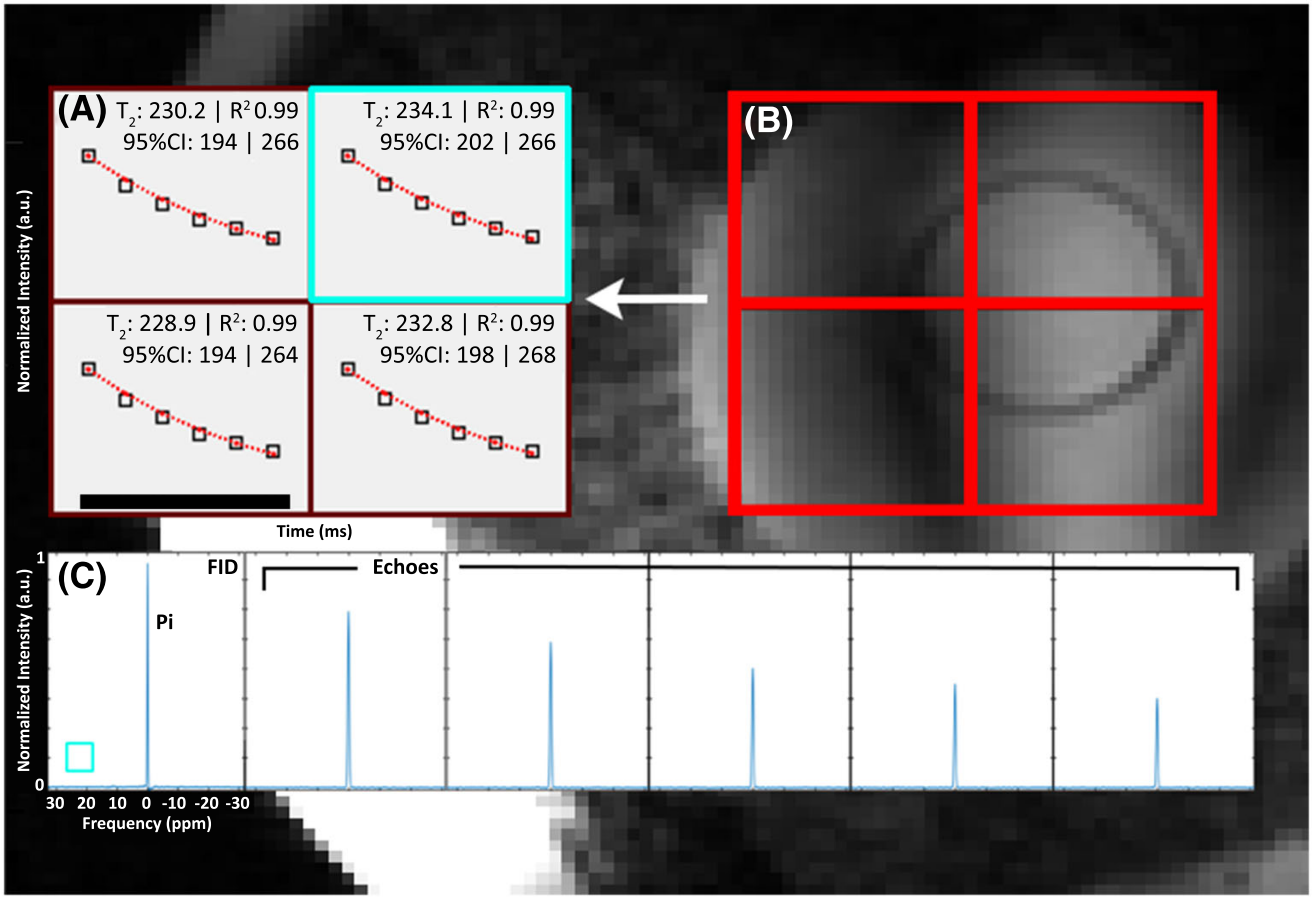
A, B, in vitro *T*_2_ measurement of P_i_ in the body‐sized phantom using the multi‐echo spectral imaging sequence (a) for each voxel in the red grid on the localizer image of the sphere (B). The normalized maximum peak value for the FID and each echo plus the corresponding fit are denoted as black squares and a red dotted line respectively. Average *T*_2_ over all four voxels for the P_i_ contained in the small sphere was 232 ± 35 ms. C, the spectra of the FID and five echoes for the single voxel highlighted by the blue square, acquired using the ^31^P dual coil receiver in combination with the ^31^P body coil. The frequency scaling shown for the *x*‐axis of the FID is equal for all other echoes

**Figure 5:**
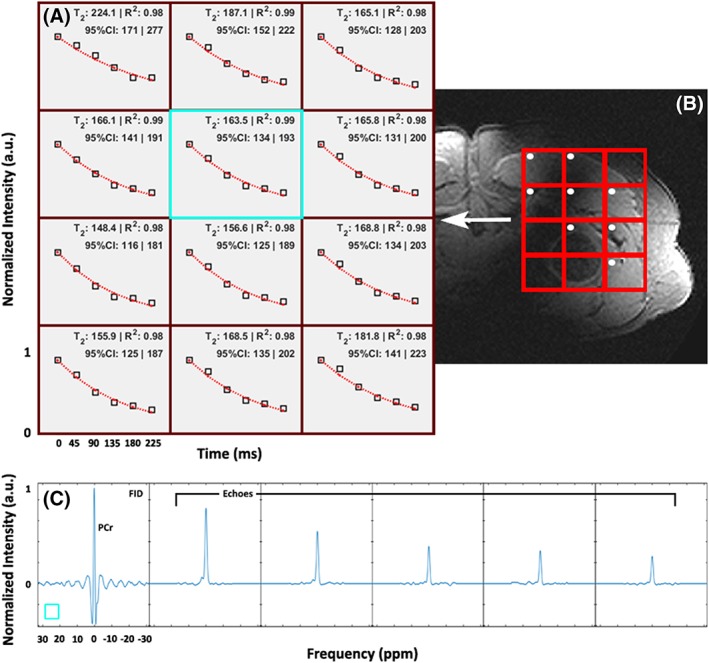
A, B, in vivo *T*_2_ fits of PCr using the MESING data from a single volunteer in voxels corresponding to the gluteal muscles (A) as shown by the red grid in the *T*_1_‐weighted localizer image (B). Normalized maximum peak value for the FID and each echo are denoted as open squares and the red dotted lines represent the fit. Echo times applicable to all voxels are shown in the bottom left. Average *T*_2_ from all voxels with high muscle tissue content, denoted by the white dots, was 177 ± 35 ms. C, spectra of the FID and five echoes for the voxel highlighted by the blue square, acquired using the ^31^P dual coil receiver in combination with the ^31^P body coil. The frequency scaling shown for the *x*‐axis of the FID is equal for all other echoes

**Figure 6:**
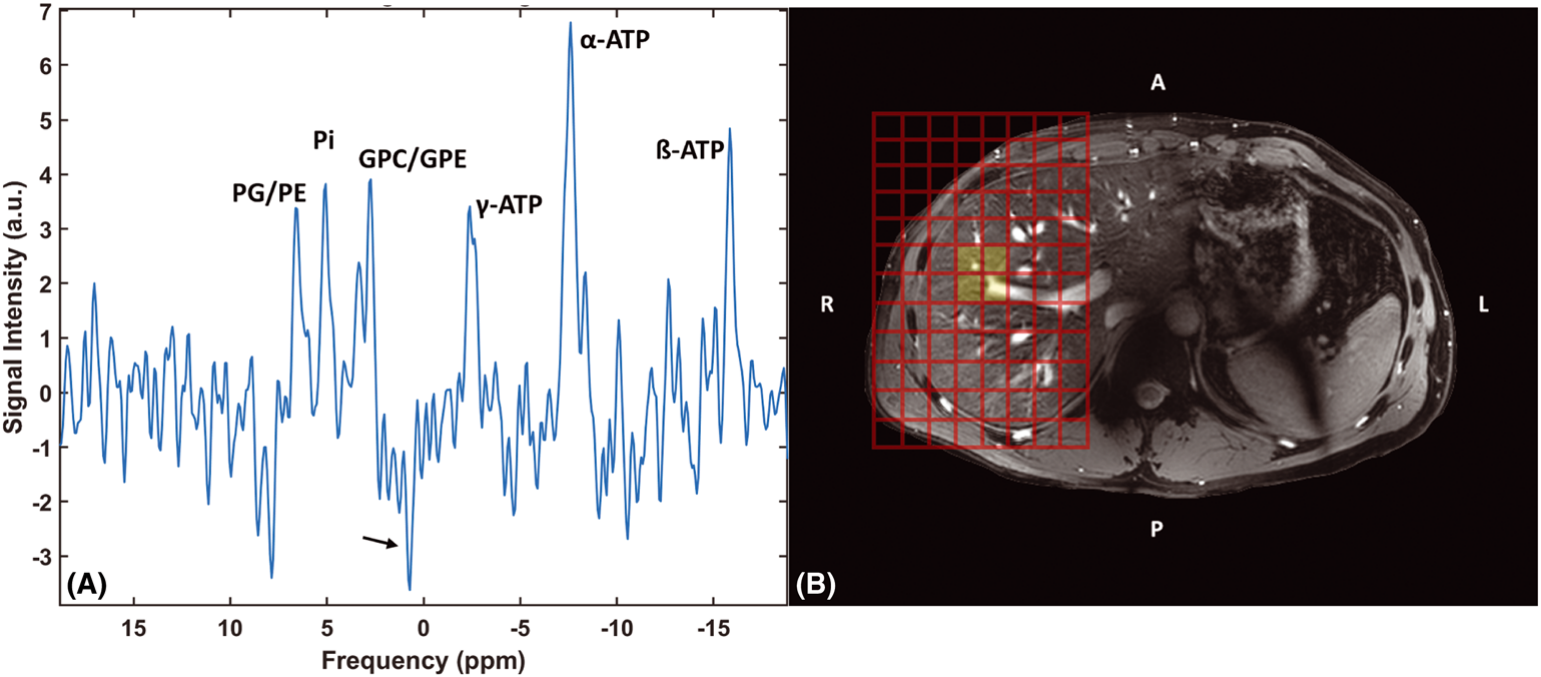
A, B, liver spectrum (A) after averaging all liver voxels from the 3D CSI protocol shown in the localizer image (B). Data were acquired with the ^31^P whole‐body transmit coil in combination with the ^31^P receiver coils in quadrature mode. Metabolite peaks of PME, P_i_, PDE and the three ATP resonances are denoted. The arrow points to the opposite phased PCr resulting from residual signal contamination from the muscles

The integrated body coil in combination with the quadrature ^31^P receive loops showed high SNR ^31^P MRSI (3.9 for PME to 82 for PCr), as shown in the spectra in Figures 3, 4, 5, 6. *B*_0_ shimming and partial volume effects were suboptimal over such large field of view, with a measured line width of 0.20 ppm before apodization. *T*_1_ weighting is apparent from the relative decrease of PCr and increase of α‐ and γ‐ATP resonances in Figure 3C compared with Figure 3D. In addition, the β‐ATP peak is decreased and the PDE peak shows a similar but minor decrease. An increase of the P_i_ signal is noticed in Figure 3D compared with Figure 3C.

The MESING data were acquired well within SAR limits and with sufficient SNR to allow *T*_2_ fitting (Figures 4 and 5). The MESING refocusing 180° composite block pulse used 15 μT and was 4.26 ms long, which compared with the 100 μT adiabatic RF pulse of 8 ms of the AMESING sequence used in the breast by van der Kemp et al resulted in an 83‐fold reduced effective *B*_1_ integral. The in vitro average *T*_2_ of P_i_ in the phantom using the MESING method was 232 ± 35 ms (Figure 4) and the *T*_2_ of PCr from a single volunteer measured 177 ± 35 ms (Figure 5). Base‐line roll artifact is visible in the FID in Figures 4C and 5C resulting from equal data processing of FID and echoes by correcting for the bandwidth difference. As the ^1^H antennas are inherently decoupled from the ^31^P coils, adequate ^1^H MR images for localization could be obtained, shown by the proton images in Figures 4B and 5B.

Averaging four local liver voxels from the 3D CSI protocol resulted in sufficient SNR to discriminate PD, PC, GPC, GPE, P_i_ and ATP resonances (Figure 6). The yellow voxels in Figure 6B indicate the voxels' origin, and a residual opposite phased PCr peak, denoted by an arrow, is visible in the spectrum.

## 4 DISCUSSION

Multiple, SAR demanding, body oriented ^31^P MRSI methods were explored successfully using the fully integrated ^31^P whole‐body coil at 7 T. Power calibrations of the homebuilt birdcage coil in multiple volunteers showed consistent performance with 30% inter‐subject variability of coil load. Metabolic information from the gluteus maximus and the full liver was acquired, and the multi‐echo CSI method was successfully implemented. Simultaneous use of the ^31^P receiver coils with the ^1^H transceiver antennas preserved volunteer comfort, as more freedom was experienced due to the lack of additional enclosing hardware that normally a ^31^P transmit coil would require. The ^31^P whole‐body coil with uniform excitation in the body enabled the use of low demand SAR, conventional rectangular RF pulses instead of the high energy adiabatic RF pulses required with conventional surface coils for achieving homogeneous excitations. This decreases overall SAR, increasing the number of possible RF excitations per scan time to permit reduction of acquisition duration by decreasing *T*_R_ or even the use of high flip angles, in ^31^P MRSI.

*B*_1_^+^ field homogeneity was assessed from designs by Löring as our design is merely scaled to the bore size. The homogeneity of the insert was shown from the use of *B*_1_ maps from 3 T proton MRI, which have identical diameter and coil layout to the present ^31^P body coil and are tuned to almost the same frequency.^20^

Liver spectra were acquired over a large field of view and with minimal signal contamination by positioning the volunteer in the right decubitus position, weighted *k*‐space acquisition and small voxel size. Increasing the number of sample averages regained SNR per voxel. This allowed discrimination of the mono‐ and di‐esters PE, PC and GPC, GPE respectively.

MESING was validated on a phantom with P_i_, as the average *T*_2_ of 232 ms found corresponded to the *T*_2_ of the body‐sized phantom measured with the conventional AMESING sequence from van der Kemp et al (data not shown).^8^ The in vivo application of MESING showed an average *T*_2_ of PCr in the gluteal muscle of 177 ms ± 35 ms, which is comparable to the reported *T*_2_ value of PCr in the calf muscle of around 217 ± 14 ms.^8^, ^24^ Note that the *T*_2_ value measured by Bogner et al^24^ is an average for seven volunteers, where individual physiological differences between volunteers are averaged out, while our measured value in the gluteal muscle is an average from multiple voxels for just one volunteer, without averaging out possible physiological differences. Another possible cause for a difference in *T*_2_ is sub‐optimal refocusing pulses caused by imperfect power adjustments; however, the flip angle sweep in Figure 2 shows little variance between subjects, making it less likely to be the source of a lower *T*_2_. A difference in physiology of the gluteal muscle compared with the calf muscle could result in a slightly higher chemical exchange rate between PCr and ATP, which leads to a lower *T*_2_.^18^, ^19^, ^25^

The *T*_2_ relaxation property of the metabolites acquired with MESING provided a higher information density from the ^31^P spectra compared with a conventional MRSI experiment. Because metabolite specific MR properties are available, the signal of each individual metabolite of interest can be corrected for *T*_2_ blurring during acquisition, subsequently favoring SNR or used as a new contrast for each metabolite. This increases diagnostic significance and allows for new research in molecular dynamics and tissue environments. It can also be of interest to areas where *B*_0_ shimming can be difficult, as the reduced spectral SNR caused by static *B*_0_ inhomogeneities could be regained using the MESING method. In conventional proton MRI, *T*_2_ is an important biomarker to discriminate tumor from healthy tissue, aiding in diagnosis and disease prognosis. However, MRI focuses on morphological changes whilst metabolic changes occur prior to any observable structural alterations, creating opportunities for MRSI.^26^, ^27^ *T*_2_ contrast in MRSI, however, is still not available in the clinic but may increase insight into diseases when used as a biomarker including relaxation information for each metabolite separately. Though quantification of metabolite concentrations requires no transverse or longitudinal relaxation weighting, it has recently been shown by van der Kemp et al that shortening of the transverse relaxation time of P_i_ can be used as a biomarker in breast cancer spectroscopy.^18^

In our *T*_1_‐weighted ^31^P MRSI focusing on P_i_, we choose two *T*_R_ and flip angle combinations, which remained close to and deviated from the optimal Ernst angle condition for cytosolic P_i_, allowing for *T*_1_ weighting with the latter condition. Other metabolites are *T*_1_ weighted in both situations; however, the weighting is amplified for PCr, PME and PDE, with *T*_1_ relaxation rates of the order of several seconds (≥3.1 s), whereas the optimal Ernst angle condition is almost met for the β‐ and γ‐ATP resonances, with *T*_1_ relaxation rates of around 1800 ms in the high flip angle experiment.^24^ As such, SNR remained high and *T*_1_ contrast fair, as can be seen by the increased peaks of γ‐ and α‐ATP resonances and decrease of PCr. The observed decrease of the β‐ATP peak in our measurements can be explained by the decreased excitation bandwidth at higher flip angle. Figure 3C and 3D shows minor change between the two P_i_ peaks with respect to the noticeable decrease of PCr. Theoretically this could suggest an increase in signal contribution from intra‐mitochondrial P_i_.

The adaptation of the RF pulses to operate at 15 μT rather than 100 μT comes at the cost of a lower pulse bandwidth. The implemented composite refocusing pulse used in MESING has a bandwidth of less than 240 Hz. However, setting the carrier frequency to PCr resulted in higher SNR compared with lower concentration metabolites and allowed validation of the adapted sequence in vivo. The use of multi‐band RF pulses may be considered to broaden the bandwidth, or, in analogy with multi‐slice TSE, rather than exciting slices sequentially within the *T*_R_, multiple narrow band excitations could be combined to cover the entire spectrum within the same scan time. As RF power deposition with conventional RF pulses is substantially decreased, more alternative pulse sequences, similar to pulses used in proton MRI, can be applied.

In our study we have used a two‐channel receiver array, merely to demonstrate the feasibility of body‐oriented ^31^P MRSI. When expanding the receiver array to a total of 16 or 32 elements, as already shown by Valkovič et al, full body coverage can be obtained.^28^ Combined information from multiple coils around the body could increase field of depth, as SNR and subsequently sensitivity can be regained by strategic coil combination methods such as whitened singular value decomposition.^29^ The space requirements for such setup may be comparable to those of conventional clinical MRI receiver arrays, where 16‐channel body arrays are being used on a regular basis. The ^31^P receivers, as demonstrated here, can be merged with the relatively thin dipole antennas as can be seen in Figure 1A and aB, without efficiency losses.^20^

While we have shown that a uniform transmit field with highly sensitive reception fields can be achieved with the whole‐body coil and merged with a ^1^H imaging setup, care must be taken in optimizing scan protocols for motion artifacts and *B*_0_ shimming. The MESING sequence can be used to regain SNR loss caused by imperfect *B*_0_ shimming, but will not compensate for dynamic *B*_0_ changes, nor will it improve line widths. Real time dynamic shimming using training sets or field cameras can improve spectral resolution, yet these require even more additional hardware.^30^, ^31^, ^32^ Another alternative could be to use rapid MRSI techniques that include (compressed) SENSE, EPI or spiral readouts, in principle facilitating single shot MRSI acquisitions, where each shot can be frequency aligned prior to averaging.^22^ Even without any of these techniques, spectral ^31^P resolution at 7 T was 0.20 ppm, sufficient to discriminate the metabolites of interest in the human body. However, increased spectral resolution would allow improved discrimination of for instance the two P_i_ species.

## 5 CONCLUSION

The homebuilt fully integrated ^31^P body coil allowed ^31^P MRS methods to be explored that would have been SAR demanding with surface coils. Without sacrificing bore space, the improved hardware allowed full liver coverage ^31^P MRSI, and a multi‐echo sequence, with inherently lower SAR, was successfully implemented for use in the body. The latter technique, though with improvements, allows for further research into new approaches in MRS biomarkers and additional metabolite specific information.

## References

[1] Hwang J‐H , Choi CS . Use of in vivo magnetic resonance spectroscopy for studying metabolic diseases. Exp Mol Med. 2015;47(2):e139 10.1038/emm.2014.101 25656949 PMC4346484

[2] Hwang J‐H , Stein DT , Barzilai N , et al. Increased intrahepatic triglyceride is associated with peripheral insulin resistance: in vivo MR imaging and spectroscopy studies. Am J Physiol Endocrinol Metab. 2007;293(6):E1663‐E1669. 10.1152/ajpendo.00590.2006 17911339

[3] Rider OJ , Francis JM , Ali MK , et al. Effects of catecholamine stress on diastolic function and myocardial energetics in obesity. Circulation. 2012;125(12):1511‐1519. 10.1161/CIRCULATIONAHA.111.069518 22368152

[4] Valkovič L , Chmelík M , Krššák M . *In‐vivo* ^31^P‐MRS of skeletal muscle and liver: a way for non‐invasive assessment of their metabolism. Anal Biochem. 2017;529:193‐215. 10.1016/j.ab.2017.01.018 28119063 PMC5478074

[5] Klomp DWJ , van de Bank, BL , Raaijmakers A , Korteweg MA , Possanzini C , Boer VO , van de Berg CA , van de Bosch MA , Luijten PR ^31^P MRSI and ^1^H MRS at 7 T: initial results in human breast cancer. NMR Biomed 2011;24(10):1337–1342. 10.1002/nbm.1696. 21433156

[6] van der Kemp WJ , Stehouwer BL , Luijten PR , van den Bosch MA , Klomp DW . Detection of alterations in membrane metabolism during neoadjuvant chemotherapy in patients with breast cancer using phosphorus magnetic resonance spectroscopy at 7 tesla. SpringerPlus. 2014;3(1):634 10.1186/2193-1801-3-634 25932360 PMC4409619

[7] Purvis LAB , Clarke WT , Valkovič L , et al. Phosphodiester content measured in human liver by in vivo ^31^P MR spectroscopy at 7 tesla. Magn Reson Med. 2017;78(6):2095‐2105. 10.1002/mrm.26635 28244131 PMC5697655

[8] van der Kemp WJM , Boer VO , Luijten PR , Stehouwer BL , Veldhuis WB , Klomp DWJ . Adiabatic multi‐echo ^31^P spectroscopic imaging (AMESING) at 7 T for the measurement of transverse relaxation times and regaining of sensitivity in tissues with short *T* _2_* values. NMR Biomed. 2013;26(10):1299‐1307. 10.1002/nbm.2952 23553945

[9] Negendank W . Studies of human tumors by MRS: a review. NMR Biomed. 1992;5(5):303‐324. 10.1002/nbm.1940050518 1333263

[10] Aboagye EO , Bhujwalla ZM . Malignant transformation alters membrane choline phospholipid metabolism of human mammary epithelial cells. Cancer Res. 1999;59(1):80‐84. 9892190

[11] Glunde K , Jie C , Bhujwalla ZM . Molecular causes of the aberrant choline phospholipid metabolism in breast cancer. Cancer Res. 2004;64(12):4270‐4276. 10.1158/0008-5472.CAN-03-3829 15205341

[12] Park JM , Park JH . Human *in‐vivo* 31P MR spectroscopy of benign and malignant breast tumors. Korean J Radiol. 2001;2(2):80 10.3348/kjr.2001.2.2.80 11752975 PMC2718106

[13] Runge JH , van der Kemp WJM , Klomp DWJ , Luijten PR , Nederveen AJ , Stoker J . 2D AMESING multi‐echo ^31^P‐MRSI of the liver at 7T allows transverse relaxation assessment and *T* _2_‐weighted averaging for improved SNR. Magn Reson Imaging. 2016;34(2):219‐226. 10.1016/j.mri.2015.10.018 26597833

[14] Steen RG . Response of solid tumors to chemotherapy monitored by *in vivo* ^31^P nuclear magnetic resonance spectroscopy: a review. Cancer Res. 1989;49(15):4075‐4085. 2663140

[15] Ng TC , Grundfest S , Vijayakumar S , et al. Therapeutic response of breast carcinoma monitored by ^31^P MRS *in situ* . Magn Reson Med. 1989;10(1):125‐134. 10.1002/mrm.1910100112 2547134

[16] Semmler W , Gademann G , Bachert‐Baumann P , Zabel HJ , Lorenz WJ , van Kaick G . Monitoring human tumor response to therapy by means of P‐31 MR spectroscopy. Radiology. 1988;166(2):533‐539. 10.1148/radiology.166.2.3336731 3336731

[17] Kan HE , Klomp DWJ , Wong CS , et al. *In vivo* ^31^P MRS detection of an alkaline inorganic phosphate pool with short T1 in human resting skeletal muscle. NMR Biomed. 2010;23(8):995‐1000. 10.1002/nbm.1517 20878975 PMC3856567

[18] van der Kemp WJM , van der Velden TA , Schmitz AM , et al. Shortening of apparent transverse relaxation time of inorganic phosphate as a breast cancer biomarker. NMR Biomed. 2018:e4011 10.1002/nbm.4011 30311703 PMC6899594

[19] van der Kemp WJM , Klomp DWJ , Wijnen JP . ^31^P T_2_s of phosphomonoesters, phosphodiesters, and inorganic phosphate in the human brain at 7T. Magn Reson Med. 2018;80(1):29‐35. 10.1002/mrm.27026 29215148 PMC5900879

[20] Löring J , van der Kemp WJM , Almujayyaz S , van Oorschot JWM , Luijten PR , Klomp DWJ . Whole‐body radiofrequency coil for ^31^P MRSI at 7 T. NMR Biomed. 2016;29(6):709‐720. 10.1002/nbm.3517 27037615

[21] Raaijmakers AJE , Italiaander M , Voogt IJ , et al. The fractionated dipole antenna: a new antenna for body imaging at 7 tesla. Magn Reson Med. 2016;75(3):1366‐1374. 10.1002/mrm.25596 25939890

[22] Pohmann R , von Kienlin M , Haase A . Theoretical evaluation and comparison of fast chemical shift imaging methods. J Magn Reson. 1997;129(2):145‐160. 10.1006/jmre.1997.1245 9441879

[23] Chmelik M , Považan M , Krššák M , et al. *In vivo* ^31^P magnetic resonance spectroscopy of the human liver at 7 T: an initial experience. NMR Biomed. 2014;27(4):478‐485. 10.1002/nbm.3084 24615903

[24] Bogner W , Chmelik M , Schmid AI , Moser E , Trattnig S , Gruber S . Assessment of ^31^P relaxation times in the human calf muscle: a comparison between 3 T and 7 T in vivo. Magn Reson Med. 2009;62(3):574‐582. 10.1002/mrm.22057 19526487

[25] Ren J , Sherry AD , Malloy CR . ^31^P‐MRS of healthy human brain: ATP synthesis, metabolite concentrations, pH, and *T* _1_ relaxation times. NMR Biomed. 2015;28(11):1455‐1462. 10.1002/nbm.3384 26404723 PMC4772768

[26] Cheng W , Ping Y , Zhang Y , Chuang K‐H , Liu Y . Magnetic resonance imaging (MRI) contrast agents for tumor diagnosis. J Healthc Eng. 2013;4(1):23‐46. 10.1260/2040-2295.4.1.23 23502248

[27] Chung WJ , Chung HW , Shin MJ , et al. MRI to differentiate benign from malignant soft‐tissue tumours of the extremities: a simplified systematic imaging approach using depth, size and heterogeneity of signal intensity. Br J Radiol. 2012;85(1018):e831‐e836. 10.1259/bjr/27487871 22553293 PMC3474004

[28] Valkovič L , Dragonu I , Almujayyaz S , et al. Using a whole‐body ^31^P birdcage transmit coil and 16‐element receive array for human cardiac metabolic imaging at 7T. PLoS ONE. 2017;12(10):e0187153 10.1371/journal.pone.0187153 29073228 PMC5658155

[29] Rodgers CT , Robson MD . Receive array magnetic resonance spectroscopy: whitened singular value decomposition (WSVD) gives optimal Bayesian solution. Magn Reson Med. 2010;63(4):881‐891. 10.1002/mrm.22230 20373389

[30] Barmet C , Zanche ND , Pruessmann KP . Spatiotemporal magnetic field monitoring for MR. Magn Reson Med. 2008;60(1):187‐197. 10.1002/mrm.21603 18581361

[31] Boer VO , van de Bank BL , van Vliet G , Luijten PR , Klomp DWJ . Direct *B* _0_ field monitoring and real‐time *B* _0_ field updating in the human breast at 7 tesla. Magn Reson Med. 2012;67(2):586‐591. 10.1002/mrm.23272 22161736

[32] Dietrich BE , Brunner DO , Wilm BJ , et al. A field camera for MR sequence monitoring and system analysis. Magn Reson Med. 2016;75(4):1831‐1840. 10.1002/mrm.25770 25975352

